# A Protein Microarray-Based Investigation of Cerebrospinal Fluid Reveals Distinct Autoantibody Signature in Low and High-Grade Gliomas

**DOI:** 10.3389/fonc.2020.543947

**Published:** 2020-12-22

**Authors:** Nikita Gahoi, Parvez Syed, Saket Choudhary, Sridhar Epari, Aliasgar Moiyadi, Santosh G. Varma, Mayuri N. Gandhi, Sanjeeva Srivastava

**Affiliations:** ^1^Wadhwani Research Center for Biosciences and Bioengineering, Department of Biosciences and Bioengineering, Indian Institute of Technology Bombay, Mumbai, India; ^2^Centre for Research in Nanotechnology and Sciences, Indian Institute of Technology Bombay, Mumbai, India; ^3^Inme Oy, Turku, Finland; ^4^Department of Chemical Engineering, Indian Institute of Technology Bombay, Mumbai, India; ^5^Molecular and Computational Biology, Department of Biological Sciences, University of Southern California, Los Angeles, CA, United States; ^6^Department of Pathology, Tata Memorial Centre, Mumbai, India; ^7^Neurosurgical Services, Department of Surgical Oncology, Tata Memorial Centre and Homi Bhabha National Institute, Mumbai, India; ^8^Deptartment of Biochemistry, Grant Govt. Medical College and Sir JJ Group of Hospitals, Mumbai, India; ^9^Department of Biochemistry, BJ Medical College and Sassoon Hospital, Pune, India

**Keywords:** cancer biomarkers, glioma, cerebrospinal fluid, protein microarray, *Glioblastoma multiforme*, autoantibodies

## Abstract

Gliomas are one of the most aggressive primary brain tumors arising from neural progenitor cells. Delayed diagnosis, invasive biopsy, and diagnostic challenges stems the need for specific, minimally-invasive, and early diagnostic biomarkers. Tumor-associated (TA) autoantibodies are measurable in the biofluids long before the onset of the symptoms, suggesting their role in early diagnosis and clinical management of the patients. In the current study, cerebrospinal fluid (CSF) samples from patients with low-grade glioma (LGG) and the *Glioblastoma multiforme* (GBM) that characterizes advanced disease were compared with healthy control samples to identify putative TA autoantibodies, using protein microarrays. The CSF samples from LGGs (n = 10), GBM (n = 7) were compared with the control CSF samples (n = 6). Proteins showing significant antigenic response were cross-verified. Proteins NOL4 (a cancer-testis antigen) and KALRN showed an antigenic response in the CSF of GBM patients, whereas, UTP4 and CCDC28A showed an antigenic response in low grade gliomas when compared with the control samples. TA autoantibodies identified in this study from the CSF of the patients could supplement current screening modalities. Further validation of these TA autoantibodies on a larger clinical cohort could provide cues towards relevance of these proteins in early diagnosis of the disease.

## Introduction

Gliomas are one of the most aggressive primary brain tumors characterized by high morbidity and mortality rates due to their localization, invasive, and heterogeneous nature (1). Based on the histological characteristics, gliomas were traditionally classified into astrocytic, oligodendroglial, or ependymal tumor and were further assigned WHO grades I–IV, indicating the degree of invasiveness and malignancy. The advancement in genomic, transcriptomic and epigenetic profiling techniques has improvised the molecular understanding of gliomas and led to reclassification of these tumors based upon tumor histories, response to treatment and clinical outcomes (2). The 2016 WHO classification, grouped the diffused low-grade gliomas (WHO grade II) and intermediate-grade gliomas (WHO grade III) as low-grade gliomas (LGGs). These LGGs are further sub-divided based on the molecular markers like mutations in IDH, ATRX, TP53, and co-deletion of 1p and 19q arms of chromosomes (3). Pilocytic astrocytomas (WHO Grade I), the most common type of glioma in children, are molecularly distinct from adult gliomas. These are characterized by favorable prognosis, circumscribed growth and frequently carry BRAF gene mutations or fusion. Grade IV gliomas also known as *Glioblastoma multiforme* (GBM) are most aggressive among gliomas and characterized by cells having high mitotic rates, nuclear atypia with adjoining areas of new vessel formation and necrosis. GBMs are highly infiltrative, favor growth surrounding glial cells by suppressing immune response. GBMs are further classified into primary and secondary GBMs. Primary GBMs develop *de novo* and are highly aggressive with a survival period of less than 2 years, whereas secondary GBMs evolves gradually from lower grade gliomas and have a better prognosis than primary GBMs. Primary and secondary GBMs are histopathologically indistinguishable, however they harbor different molecular alterations (4).

Tumorigenesis is a result of accumulation of mutations in cells over time leading to production of aberrant biomolecules that may be antigenic. These atypical biomolecules are commonly referred to as tumor associated (TA) antigens and evoke immunological response resulting in production of antibodies against self-proteins, also known as TA autoantibodies. TA autoantibodies have characteristics of antibodies like specificity, production in large quantities, long half-lives and circulation in biofluids (5) and have been reported to be diagnosed five years before the development of clinical symptoms (6, 7) making them promising biomarker candidates in cancers. Till date, several TA autoantibodies have been reported in sera of cancer patients. However, the presence of blood-brain barrier in central nervous system (CNS) restricts the entry of these autoantibodies into the patient sera. The cerebrospinal fluid in the CNS acts as an amalgamation of the transported biological substances, the waste and toxic substances excreted from the brain. This makes the CSF an invaluable source of biomarkers for diagnosis, prognosis of the course of CNS disease, and also as a predictive biofluid in the presymptomatic state.

High density protein microarrays provide an important platform for antigen display (8) and allow assessment of thousands of tumor antigens simultaneously with a minimal requirement of biological samples. These arrays have been applied to study immune response against several auto-immune diseases and cancers. To screen the presence of TA autoantibody signature in CSF of the glioma patients, protein microarray (ProtoArray^©^ V5.0) containing ~9,000 human recombinant proteins has been used. To the best of our knowledge this is the first pilot experiment where CSF has been used to screen the presence of TA autoantibodies in the glioma patients.

## Materials and Methods

### Patient CSF Sample Information

CSF samples used in this study were collected from Advanced Centre for Treatment Research and Education in Cancer (ACTREC), and Tata Memorial Hospital, Mumbai, India. The study was approved by the TMC-ACTREC-Institutional Review Board (ACTREC-TMC IEC No. 15). The samples were collected after prior written consent. CSF was collected from the radiologically verified glioma patients undergoing surgery. CSF samples from 17 individuals with gliomas and 6 control patients (without any history of any intracranial infection or surgery) were used for autoantibody screening. The aliquots of the collected samples were stored at -80°C until further use. The CSF samples used in the study were classified as low-grade glioma LGGs (n = 10), GBM (n = 7), and control subjects (n = 6). Detailed information about the samples is provided in **Supplementary Tables 1** and **2**.

### Microarray Analysis

**Figure 1:**
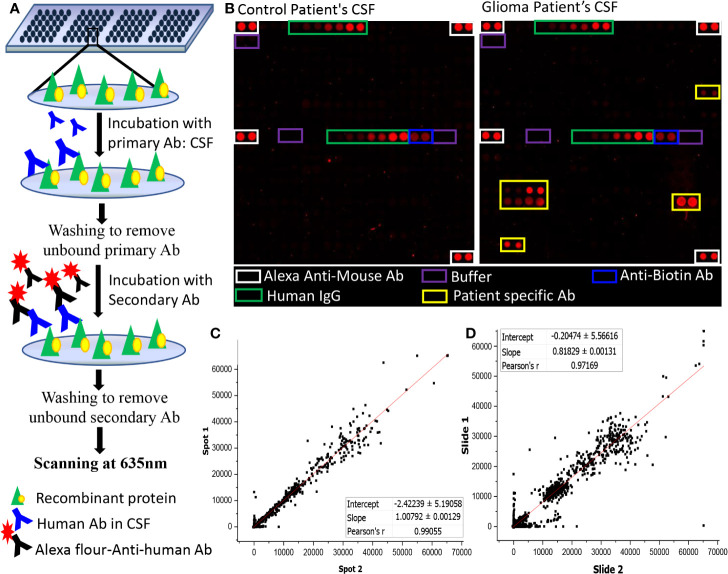
Autoantibody screening using protoarray. **(A)** Schematic representation of the experimental workflow; **(B)** Image of two sub-arrays printed on a protoarray slide, left panel represents the location of control spots on the subarray and right panel represents the location of control spots on the subarray along with signal response due to patient specific Ab; **(C, D)** Graph representing **(C)** intra- chip reproducibility, spot intensities of duplicate protein spots from same array; **(D)** Inter-chip reproducibility of slides, intensities of protein spots from two different protoarrays processed using two different samples were plotted.

Human protein microarray (ProtoArray^©^ V5.0), were purchased from Invitrogen, Carlsbad, CA. Each ProtoArray protein array contains around 9,000 GST-tagged full-length human proteins in duplicates. These full-length N-terminal GST fusion proteins are expressed using Baculovirus expression system and were purified under non-denaturing conditions to maintain the protein integrity and function. The purified proteins are then printed on an ultrathin layer of nitrocellulose coated glass slides under temperature and humidity-controlled environment. Each block on a ProtoArray slide contains positive (Alexa Flour Ab, Human IgG, Anti-human IgG, and V5 control protein) and negative (Buffer, BSA, and GST) control spotted in duplicates (**Figure 1B**).

### Autoantibody Screening

The ProtoArray slides were blocked using SuperBlock (Pierce) at 4°C for 1 h with gentle shaking followed by washing with PBST buffer (4 x 5 min) at room temperature. Each slide was probed with single CSF sample and a dilution of 1:4 CSF in blocking buffer (25 µl of CSF in 75 µl of blocking buffer) was incubated onto the slides overnight using coverslips. The slides were then washed with PBST buffer (4X5 mins), rinsed in distilled water and dried at 900 rpm for 2 min. A dilution of 1:5,000 of Cy5-labelled Goat Anti-Human Ab in blocking buffer was used as secondary Ab and the slides were incubated for an hour at room temperature on the shaker. After which the slides were washed with PBST (4 times at 5 min interval each), rinsed with distilled water and centrifuged at 4°C for 2 min at 900 rpm. Scanning of the dried slides was performed at 635 nm at 600 PMT gain using GenePix 4000B Microarray Scanner (Molecular Devices) (**Figure 1A**).

### Statistical Analysis for the Identification of Autoantibody Signatures

Images obtained after scanning the microarray slides were processed using GenePix Pro7 (Molecular Devices) software. The acquired data, that is, the median pixel intensity values obtained for each protein spot was analyzed using two different methods. In the first method, median pixel intensity (F635) subtracted by local background (B635) for each spot was considered for analysis (median F635-B635). A base cut-off of 60 was set to replace all the negative values and values below 60. Sample specific normalization was performed by subtracting the median value of multiple buffer spot present in the array to the intensity of protein spots that are printed throughout the chip (9). The normalized signal intensities of all the duplicate spots were averaged and log2 transformed, this value was further used for statistical analysis using Metaboanalyst 4.0 (10). Two-tailed *t*-statistics was applied to identify the proteins with antigenic potential. Further, the p-values were adjusted for false discovery using Bonferroni correction. To cross-examine the data obtained, dot plots for all the significant proteins (raw p-value <0.005 and absolute FC >1.2) were plotted for different comparison using GraphPad Prism (Prism v6.0, GraphPad Software Inc., La Jolla CA).

In the second method, the data was pre-processed to adjust the technical errors between the arrays, thereby adjusting the differences that did not arise biologically. “Limma” package (11) made available as a Bioconductor package (12) for R programming was used to pre-process the data. Pre-processing of the data was performed using the “neqc” function that performs both background correction and quantile normalization using a set of negative control genes. Pre-processed data was then analyzed to look for the proteins with antigenic potential. “Limma” package uses moderated *t*-statistic to test the null hypothesis that proteins are not differentially expressed between two conditions. To adjust for multiple hypothesis testing, we used, “Benjamini-Hochberg” (BH) correction. Statistically significant proteins with adjusted p-value <0.05 were sorted and the proteins with fold-change ≥ ± 1.5 were considered for further analysis. Autoantibody response for these proteins was manually verified for all the samples.

### Gene Set Enrichment Analysis

To understand the functional, molecular and sub-cellular characteristics, gene set enrichment analysis of proteins showing antigenic characteristic (p-value <0.05 and abs FC >1.2) was performed using software like DAVID (DAVID Bioinformatics Resources 6.7) (13) and Protein ANalysis THrough Evolutionary Relationships (PANTHER) (14) system version 7 (http://www.pantherdb.org).

## Results

### Quality Check of the Processed Slides

The intra-chip variation was evaluated by calculating the sample coefficient of variation (CV) between the duplicate spots using “CV” function of R programming. The CV were calculated for each slide using the raw pixel intensity (median F635-B635). The value of CV ranged from 0.14 to 0.31 with an average of 0.215 ± 0.04 **(****Supplementary Table 3****)**. Further, a graphical representation of intra-chip and inter-chip correlation is given in **Figures 1C**, **D**. The intensities of the protein spots were checked for all slides showing lower intensities in the positive control spots. The protein spots showed comparable signal intensities with protein spot on other slides, hence, none of the samples were excluded from the study.

### Autoantibody Signatures in Glioma Samples

**Figure 2:**
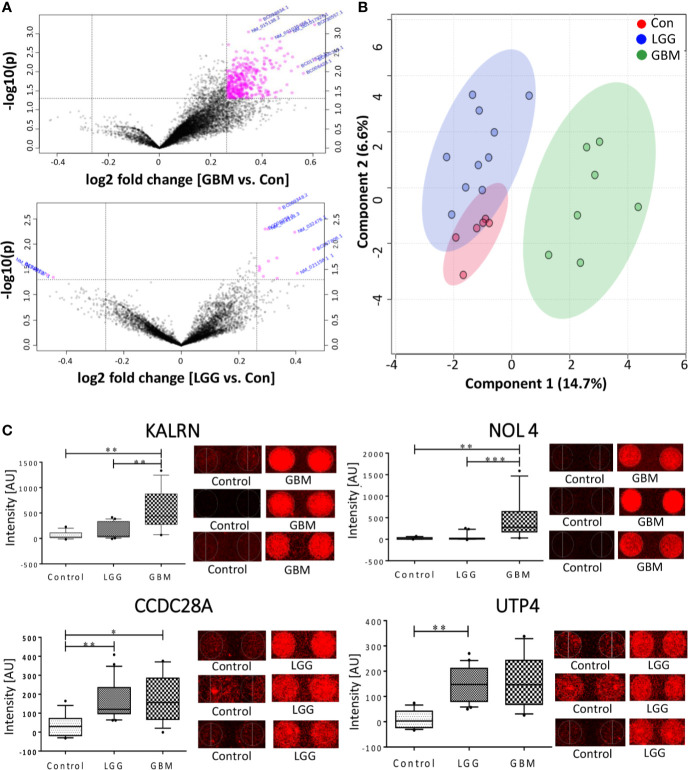
Autoantibody response in CSF of Glioma patients. **(A)** Volcano plot signifying the potentially antigenic protein in GBM and LGG samples **(B)** SPLSDA plot representing the segregation of control, LGG and GBMs, GBM showed a good segregation from control and low grade glioma; **(C)** Whiskers plot of some of the significant proteins in GBM and LGG and their respective spots on the protoarray slide. (*indicates 0.01 < p < 0.05, **indicates 0.001 < p < 0.01, ***indicates 0.0001 < p < 0.001).

The normalized data was subjected to moderated t-test and the obtained p-values were corrected using Bonferroni correction. Due to the stringent nature of the Boneferroni method, none of the proteins could pass the filtering criteria of adj. p-value <0.05. However, with a filtering criterion of raw p-value <0.05 and abs FC >1.2, a total of 15 proteins were found to show variable expression in the comparison of LGG vs. Control, of which 13 protein showed a positive fold changes while 2 proteins showed a negative fold change with respect to control samples (**Figure 2A**). The comparison of GBM with control samples yielded total 405 proteins, all of which showed a positive response in GBM samples (**Figure 2A**). Further, a cut-off of p-value less than 0.005 and abs FC >1.2, filtered a total of 33 and 3 proteins from GBM and LGG respectively when compared to control samples, list of these proteins with their fold changes and respective p-values is provided in **Supplementary Table 4**. Further, to investigate the segregating potential of these filtered proteins, partial least squares discriminant analysis (PLS-DA) and unsupervised clustering was performed. PLS-DA showed good segregation of the GBM samples from control and LGG samples whereas LGG and Control samples showed some overlap (**Figure 2B**). The unsupervised clustering revealed that the GBM samples clustered together but there was a significant overlap between LGG and Control samples (**Supplementary Figure 1****)**. These results revealed the presence of TA autoantibodies in the CSF of GBM samples is much higher as compared to low grade glioma samples. The data was further cross-validated by plotting the raw intensity values of the duplicate spots for all the proteins passing the filtering criteria of raw p-value <0.005 and FC >1.2 (**Supplementary Figure 2**).

In the other method, pre-processing of the data was performed using “neqc” function that performs both background correction and quantile normalization using the negative control spots. The pre-processed data seemed to be median centric, thereby excluding any possible technical variances and was used for further analysis. The list of differentially expressed proteins was further subjected to moderated t-statistic and “Benjamini-Hochberg” (BH) correction. The criteria of adj. p-value <0.05 and absolute FC >1.5 was applied and proteins passing the criteria were further considered to be showing an immunogenic response. In a comparison of low-grade glioma and control, 2 proteins viz., U3 small nucleolar RNA-associated protein 4 homolog (UTP4) and Coiled-Coil domain containing 28A (CCDC28A) showed a significant response. The comparison between GBM vs. control resulted in 2 proteins, namely Nucleolar protein 4/Cancer/Testis Antigen 125 (NOL4) and Kalirin (KALRN), showed an immunogenic response (**Table 1**). These proteins were also present in the list obtained from the first analysis and were further cross-validated manually by looking at the individual spots on each slide. Intensities of these significant proteins are represented in form of whisker’s plot along with their respective protein spots on the protoarray slide in **Figure 2C**.

**Table 1:** List of significant proteins against which TA autoantibodies were observed in low grade glioma and *glioblastoma multiforme* samples.

Gene ID	Protein name	Uniprot ID	Log FC	FC	p-value	Adj. p-value
**Low Grade Glioma vs. Control**						
BC009348.2	U3 small nucleolar RNA-associated protein 4 homolog	UTP4	0.60	1.516	3.36E-05	0.033
BC000758.1	Coiled-Coil domain containing 28A	CCDC28A	0.619	1.536	6.80E-05	0.046
**Glioblastoma Multiforme vs. Control**						
BC000313.1	Nucleolar Protein 4	NOL4	1.356	2.56	5.78E-07	0.003
NM_007064.1	Kalirin	KALRN	1.264	2.40	6.19E-07	0.003

### Gene Set Enrichment Analysis

Bioinformatics tools like Panther and DAVID were used to characterize the list of proteins obtained from the statistical analysis using Metaboanalyst. The sub-cellular localization of 85.65% of these proteins was cytoplasmic, organelle-based or were a part of macromolecular complex system, while only 14.00% of the protein had membrane-linked or extra-cellular matrix-based origin. Most of these proteins categorized into protein modifying enzyme (19.20%), nucleic acid binding protein (13.60%), and metabolite interconversion enzyme (10.20%). Some of these proteins were associated with angiogenesis, inflammation, B cell activation pathway, signaling pathway, FGF signaling pathway, PDGF signaling pathways, Wnt and Notch signaling pathway, Ras pathway, p53 pathway and EGFR signaling pathway.

## Discussion

Gliomas are neoplasms arising from neuroepithelial tissues. Molecular alteration in the neoplasm results in production of anomalous biomolecules that evokes immune response leading to production of TA autoantibodies, against self-proteins. TA autoantibodies are highly specific and are produced against modified or amplified tumor marker, with a half-life of up to 30 days in circulation and can be quantified using routinely used platforms in the clinics. These TA autoantibodies majorly belong to IgG class of immunoglobulin and have been reported to be present in the serum of cancer patients. A study reported that the rate of diagnosis of organ-specific autoimmune neurologic disorders was clinically pertinent when paraneoplastic autoantibodies were detected in both serum and CSF of the patients with clinical suspicion (15). The presence of BBB makes CSF an invaluable source of biomarkers for diagnosis, prognosis of the course of CNS disease, and also as a predictive biofluid in the presymptomatic state.

In the current study, we have used protein microarrays, containing ~9,000 recombinant proteins to detect TA autoantibody signatures in the CSF of glioma patients. The samples size used in the study was low, which increases the risk of error, therefore, the only proteins that could pass the adj p-value cut-off of less than 0.05 and fold change greater than 1.5 were considered and have been discussed in details. From the statistical analysis, two proteins viz., Nucleolar Protein 4 (NOL4) and Kalirin (KALRN) showed a significant antigenic response in GBM patients when compared to control samples. *NOL4* is a nucleolar protein encoding gene with a predominant expression in brain and testis. Aberrant methylation of CpG islands in the *NOL4* gene promoter has been reported to be associated with 85% of the cervical cancer patients (16) and 91% of head and neck squamous cell carcinoma (HNSCC) (17). An increased expression of *NOL4* has been reported in prostate cancer patients (18) and was reported to be significantly associated with the aggressiveness of the disease (19). Stangeland et al., reported *NOL4* to be one of the 20 aberrantly expressed gene in GBMs (20). KALRN is a member of Dbl family with two unique Rho guanine nucleotide exchange factors (GEFs) and multiple spectrin-like domains. mRNA expression profiling of *KALRN* gene revealed different isoforms of Kalirin produced through alternate splicing; its expression is restricted to CNS with the highest expression seen in cerebral cortex and hippocampus. Kalirin-7 is the most abundant isoform present in adult brain that regulates maturation and maintenance of dendritic spine, post-synaptic actin dynamics, axon extension and activity dependent plasticity. Mains et al., reported over-expression of Kalirin-8 (another isoform of Kalirin) in Chinese hamster ovary cells and AtT-20 cells that resulted in rearrangement of actin cytoskeleton (21). Greenman et al., performed a systemic genome sequence profiling of 518 kinases to identify possible mutations across 210 diverse human cancers. In this study, TRIO, a paralog of Kalirin, was also sequenced. Nine mutations were found in TRIO gene of which 6 mutations were present in the catalytic domains of TRIO. An insertion mutation of A156InsP (C469-470CCC) was found in glioma samples (22).

UTP4 and CCDC28A showed significant autoantigenic response in LGGs when compared with control sample. hUTP4/CIRH1A is a human ribosome biogenesis factor which is a part of t-UTP subcomplex of ribosomal small subunit (SSU) processome required for the maturation of 18S rRNA (23, 24). Homologous missense mutation (R565W) at the C-terminus of the CIRH1A has been reported in all the patients with North American Indian Childhood Cirrhosis (NAIC) (25). *In silico* analysis reported over-expression of CIRH1A in colon and para-rectum adenocarcinomas. Knock-down of CIRH1A in RKO CRC cell lines resulted in increased apoptosis, suppressed cell proliferation and cell arrest at G1 phase (26). Yu et al., reported that CIRH1A, interacts with Cirip/HIVEP and leads to up-regulation of NF-κB element (27), an active player in human cancers (28). *CCDC28A* encodes for coiled-coil domain containing protein and is a known translocation partner of nucleoporin 98 (NUP98) in T cell acute lymphoblastic leukaemia (T-ALL). Petit et al., transduced *NUP98-CCDC28A* in primary murine hematopoietic progenitor cells and reported that *NUP98-CCDC28A* promotes self-renewable potential and proliferative capacity of myeloid progenitors through an alternative leukemogenic mechanism (29).

The gene set enrichment analysis showed that only 14.00% of proteins were associated with cell junction, membrane, extracellular matrix or extracellular region. The remaining 85.65% of the proteins were associated with cytoplasm, organelle or were a part of macromolecular complex **(****Supplementary Table 5****)**. Contrary to the serum antibodies that are produced predominantly against extra-cellular protein, TA autoantibodies are produced against intra-cellular markers. Reuschenbach et al., summarized in their review that the sub-cellular distribution of the proteins against which the TA autoantibodies is produced is 42% cytoplasmic, 26.1% nucleus associated, 21.4% membrane bound, and only 10.3% extracellular (30). Several studies have reported that the TA autoantibodies epitope could be highly conserved and bind to the functional site of the proteins inhibiting the function of antigen (31–33). Therefore, TA autoantibodies could be immunological biomarkers produced against aberrant cellular mechanism related to tumorigenesis (5, 34).

The results from our findings suggest the presence of TA autoantibodies in the CSF of glioma patients. LGGs and GBM showed autoantibody response against different set of proteins, which can be well corroborated with their distinct molecular characteristics. An enhanced presence of TA autoantibodies was observed in GBM samples as compared to the low-grade gliomas. These signature molecules can be quantified in biofluids using routine clinical platforms like ELISA and could aid in early diagnosis of disease. This was an exploratory study designed to detect the presence of TA autoantibodies in CSF of the cancer patients. Nevertheless, the small sample size used in the study possess the risk of increased rate of error, therefore the findings from the study needs to be further validated on the larger cohort.

## Conclusion

Gliomas are one of the most aggressive brain tumors and they are diagnosed at an advanced stage. Therefore, an early identification of disease with less invasive methods would help in improvising the morbidity and mortality rates. Mutations in neoplasm lead to production of atypical biomolecules that evokes immune response and leads to production of TA autoantibodies. TA autoantibodies are detectable in biofluids and their quantification platforms are already in clinical use. We investigated the presence of TA autoantibodies in the CSF of glioma patients to detect the pre-symptomatic markers indicative of diseased state. Most of the CSF samples procured from GBM patients showed the presence of TA autoantibodies against NOL4 and KALRN while low grade glioma samples showed an antigenic response against UTP4 and CCDC28A. Considering the fact that the sample size used in the study was small, these finding needs to be validated on a larger population. Detecting TA autoantibodies in cancer patients could aid in early diagnosis of disease and might provide useful insight into neoplasm initiation.

## Data Availability Statement

The original contributions presented in the study are included in the article/**Supplementary Material**. Further inquiries can be directed to the corresponding author.

## Ethics Statement

The studies involving human participants were reviewed and approved by TMH-IRB. The patients/participants provided their written informed consent to participate in this study.

## Author Contributions

NG and SS conceived the idea. NG and PS performed microarray experiments. SE, AM, and SV provided clinical samples and clinical details. NG, SC, and MG performed analysis. NG and SS contributed in manuscript preparation. All authors contributed to the article and approved the submitted version.

## Funding

This research was supported by Department of Biotechnology (DBT) (No. BT/PR14359/MED/30/916/2010) and (BT/PR4599/BRB/10/1042/2012), government of India, MHRD-UAY Project, Uchhatar Avishkar Yojana, project #34_IITB (2016) to SS and intramural funding from Tata Memorial Centre (DAE-CTC grant to AM). NG was supported by Council of Scientific and Industrial Research (CSIR) Senior Research Fellowship.

## Conflict of Interest

Author PS was employed by company Inme Oy and has no competing financial interests.

The remaining authors declare that the research was conducted in the absence of any commercial or financial relationships that could be construed as a potential conflict of interest.

## Abbreviations

BBB, Blood Brain Barrier; CCDC28A, Coiled-Coil domain containing 28A; CNS, Central Nervous System; CSF, Cerebrospinal Fluid; FDR, False Discovery Rate; GBM, *Glioblastoma Multiforme*; IDH, Isocitrate Dehydrogenase; KALRN, Kalirin; LGG, Low-Grade Glioma; NOL4, Nucleolar protein 4; TA, Tumor-associated Autoantibodies; UTP4, U3 small nucleolar RNA-associated protein 4 homolog.
