# Supplementary material for: A Protein Microarray-Based Investigation of Cerebrospinal Fluid Reveals Distinct Autoantibody Signature in Low and High-Grade Gliomas
Source: Front Oncol. 2020 Dec 22;10:543947. doi: 10.3389/fonc.2020.543947 (PMC7784397; doi:10.3389/fonc.2020.543947)
Supplement: Supplementary file 1 [file DataSheet_1.docx]

**Supplementary Table 1:** Clinicopathological details of the glioma samples used in the study.

| **S. No.** | **Age** | **Sex** | **Initial Grade** | **HPR** | **1p 19q FISH** | **IDHR132H (IHC)** | **IDHR132H (Sequencing)** | **Final Status IDHR132H** | **Revised diagnosis (WHO 2016)** | **Classification** |
| --- | --- | --- | --- | --- | --- | --- | --- | --- | --- | --- |
| 1 | 37 | F | Glioma Grade III | AOD (GIII) | Non-deleted | Negative | Mutant | Mutant | AOD - NOS (IDH mutant and 1p19q non-del) | LGG |
| 2 | 35 | M | Glioma Grade III | AOA (GIII) | ND | Positive (Mutant) | Mutant | Mutant | ODG- A, NOS, IDH mutant | LGG |
| 3 | 24 | M | Glioma Grade III | GIII | ND | ND | ND | -- | AA, NOS, WHO grade III | LGG |
| 4 | 47 | M | Astrocytoma (WHO grade II) | A (GII) | Non-deleted | Positive (Mutant) | Mutant | Mutant | Diffuse astrocytoma, IDH mutant | LGG |
| 5 | 50 | M | Oligoastrocytoma (WHO grade II) | OA (GII) | Non-deleted | Positive (Mutant) | ND | Mutant | Diffuse astrocytoma, IDH mutant | LGG |
| 6 | 51 | M | Oligoastrocytoma (WHO grade II) | OA (GII) | co-deleted | Positive (Mutant) | Mutant | Mutant | Diffuse ODG, IDH mutant, 1p19q co-del | LGG |
| 7 | 48 | M | Anaplastic astrocytoma (WHO grade III) | AA (GIII) | ND | Negative | Mutant (ATRX retained) | Mutant | AA, IDH mutant | LGG |
| 8 | 29 | M | Anaplastic astrocytoma (WHO grade III) | AA (GIII) | ND | Positive (Mutant) | ND | Mutant | AA, IDH mutant | LGG |
| 9 | 47 | M | Oligoastrocytoma (WHO grade II) | OA (GII) | Non-deleted | Positive (Mutant) | ND | Mutant | AA, IDH mutant | LGG |
| 10 | 23 | F | Anaplastic oligoastrocytoma(WHO grade III) | AOA (GIII) | Non-deleted | Positive (Mutant) | Mutant | Mutant | AA, IDH mutant | LGG |
| 11 | 52 | M | Glioblastoma (WHO grade IV) | GBM | ND | Negative | ND | NC (Mostly IDH Wild type) | GBM, NOS | GBM |
| 12 | 64 | M | Glioblastoma (WHO grade IV) | GBM | ND | Negative | ND | NC (Mostly IDH Wild type) | GBM, NOS | GBM |
| 13 | 47 | M | Glioblastoma (WHO grade IV) | GBM | ND | Negative | ND | NC (Mostly IDH Wild type) | GBM, NOS | GBM |
| 14 | 47 | M | Glioblastoma (WHO grade IV). | GBM | ND | Negative | Wild type | Wild type | GBM, IDH wt | GBM |
| 15 | 50 | F | Glioblastoma (WHO grade IV) | GBM | ND | Negative | ND | NC (Mostly IDH Wild type) | GBM, NOS | GBM |
| 16 | 56 | M | Glioblastoma (WHO grade IV) | GBM | ND | Negative | ND | NC (Mostly IDH Wild type) | GBM, NOS | GBM |
| 17 | 37 | M | Glioblastoma (WHO grade IV) | GBM | ND | Negative | ND | NC (Mostly IDH Wild type) | GBM, NOS | GBM |

AA: Anaplastic Astrocytoma; A: Astrocytoma; AOD: Anaplastic Oligodendroglioma; AOA: Anaplastic Oligoastrocytoma; GBM: Glioblastoma Multiforme; OA: Oligoastrocytoma; ODG: Oligodendroglioma; ND: Not Done; NOS: Not otherwise Specified

**Supplementary Table 2:** Clinical details of the control CSF samples used in the study.

| **S.No.** | **Age** | **Sex** | **Protein (mg%)** | **Sugar (mg%)** | **RBC/hpf** | **Neutrophils %** | **Leucocytes %** | **Clinical diagnosis** |
| --- | --- | --- | --- | --- | --- | --- | --- | --- |
| 1 | 17 | F | 60 | 61 | 3 | 10 | 90 | Idiopathic intracranial hypertension |
| 2 | 21 | M | 22 | 61 | NIL | NIL | NIL | Epilepsy |
| 3 | 28 | F | 55 | 64 | NIL | 10 | 90 | Epilepsy |
| 4 | 65 | F | 23 | 98 | 2 | 5 | 95 | Fever of unknown origin |
| 5 | 45 | F | 50 | 69 | NIL | 10 | 90 | Seizure |
| 6 | 24 | M | ND | ND | 3 | 10 | 90 | Rt. Trigeminal Neuralgia |

**Supplementary Table 3:** Coefficient of variation (CV) values of each slide to assess the intra-chip variability

| **S. No.** | **Samples** | **Coefficient of variation** |
| --- | --- | --- |
| 1 | Con_1 | 0.301 |
| 2 | Con_2 | 0.250 |
| 3 | Con_3 | 0.259 |
| 4 | Con_4 | 0.178 |
| 5 | Con_5 | 0.255 |
| 6 | Con_6 | 0.183 |
| 7 | LGG_1 | 0.311 |
| 8 | LGG_2 | 0.152 |
| 9 | LGG_3 | 0.190 |
| 10 | LGG_4 | 0.157 |
| 11 | LGG_5 | 0.198 |
| 12 | LGG_6 | 0.275 |
| 13 | LGG_7 | 0.141 |
| 14 | LGG_8 | 0.169 |
| 15 | LGG_9 | 0.200 |
| 16 | LGG_10 | 0.216 |
| 17 | GBM_1 | 0.212 |
| 18 | GBM_2 | 0.240 |
| 19 | GBM_3 | 0.225 |
| 20 | GBM_4 | 0.173 |
| 21 | GBM_5 | 0.245 |
| 22 | GBM_6 | 0.217 |
| 23 | GBM_7 | 0.196 |

**Supplementary Figure 2:** Dot plots of the proteins (p-value<0.05) showing higher intensities (FC<0.83) in control samples in comparison to low grade gliomas (LGG)

**Supplementary Table 4:** List of proteins with potential antigenic properties (raw p-value <0.005 and Abs FC>1.2) in LGG and GBM sample with respective fold changes and p-values.

| **GeneBank Accession ID** | **Uniprot ID** | **Gene Name** | **FC** | **log2(FC)** | **raw. p-value** |
| --- | --- | --- | --- | --- | --- |
| **LGG vs. Control** | | | | | |
| BC009348.2 | UTP4 | UTP4, small subunit processome component | 1.27 | 0.34 | 0.002 |
| NM_004113.3 | FGF12 | Fibroblast growth factor 12 | 1.23 | 0.30 | 0.005 |
| BC000758.1 | CCDC28A | Coiled-coil domain containing 28A | 1.22 | 0.29 | 0.005 |
| **GBM vs. Control** | | | | | |
| BC000313.1 | NOL4 | Nucleolar protein 4 | 1.52 | 0.61 | 0.005 |
| BC030557.1 | CCDC186 | Coiled-coil domain containing 186 | 1.52 | 0.61 | 0.001 |
| NM_007064.1 | KALRN | Kalirin, RhoGEF kinase | 1.43 | 0.52 | 0.004 |
| NM_001017926.1 | ZHX1 | Zinc fingers and homeoboxes 1 | 1.42 | 0.51 | 0.001 |
| NM_152723.1 | CCDC89 | Coiled-coil domain containing 89 | 1.42 | 0.50 | 0.003 |
| BC020814.1 | SUGT1P3 | SGT1 homolog, MIS12 kinetochore complex assembly cochaperone pseudogene 3 | 1.40 | 0.49 | 0.004 |
| BC030262.1 | ADAMTSL1 | ADAMTS like 1 | 1.39 | 0.48 | 0.002 |
| NM_006038.1 | SPATA2 | Spermatogenesis associated 2 | 1.39 | 0.47 | 0.002 |
| NM_152676.1 | FBXO15 | F-box protein 15 | 1.38 | 0.47 | 0.002 |
| NM_000987.2 | RPL26 | Ribosomal protein L26 | 1.37 | 0.46 | 0.003 |
| NM_001039468.1 | MARK2 | Microtubule affinity regulating kinase 2 | 1.36 | 0.44 | 0.001 |
| NM_032476.1 | MRPS6 | Mitochondrial ribosomal protein S6 | 1.34 | 0.42 | 0.002 |
| NM_005855.1 | RAMP1 | Receptor activity modifying protein 1 | 1.34 | 0.42 | 0.004 |
| NM_175571.2 | GIMAP8 | GTPase, IMAP family member 8 | 1.33 | 0.41 | 0.004 |
| NM_175609.1 | ARFGAP1 | ADP ribosylation factor GTPase activating protein 1 | 1.33 | 0.41 | 0.001 |
| BC093864.1 | DLG3 | Discs large MAGUK scaffold protein 3 | 1.33 | 0.41 | 0.003 |
| NM_002787.1 | PSMA2 | Proteasome subunit alpha 2 | 1.32 | 0.40 | 0.002 |
| BC038934.1 | GJB6 | Gap junction protein beta 6 | 1.31 | 0.39 | 0.000 |
| NM_145008.1 | YPEL4 | Yippee like 4 | 1.31 | 0.39 | 0.001 |
| NM_152328.3 | ADSSL1 | Adenylosuccinate synthase like 1 | 1.30 | 0.37 | 0.005 |
| BC032382.1 | LOC146880 | Rho GTPase activating protein 27 pseudogene | 1.29 | 0.36 | 0.003 |
| NM_015138.2 | RTF1 | RTF1 homolog, Paf1/RNA polymerase II complex component | 1.27 | 0.35 | 0.001 |
| NM_207356.1 | C1orf174 | Chromosome 1 open reading frame 174 | 1.27 | 0.35 | 0.003 |
| NM_022720.5 | DGCR8 | DGCR8, microprocessor complex subunit | 1.27 | 0.35 | 0.005 |
| BC001652.2 | PWP1 | PWP1 homolog, endonuclein | 1.27 | 0.34 | 0.004 |
| BC030702.1 | MCPH1 | Microcephalin 1 | 1.26 | 0.33 | 0.003 |
| NM_002476.2 | MYL4 | Myosin light chain 4 | 1.26 | 0.33 | 0.005 |
| NM_004508.1 | IDI1 | Isopentenyl-diphosphate delta isomerase 1 | 1.25 | 0.32 | 0.004 |
| NM_032448.1 | FAM120B | Family with sequence similarity 120B | 1.23 | 0.30 | 0.003 |
| NM_000485.1 | APRT | Adenine phosphoribosyltransferase | 1.23 | 0.30 | 0.004 |
| BC018122.1 | TXNRD1 | Thioredoxin reductase 1 | 1.22 | 0.29 | 0.005 |
| NM_138448.2 | ACYP2 | Acylphosphatase 2 | 1.22 | 0.29 | 0.005 |
| NM_015963.4 | THAP4 | THAP domain containing 4 | 1.21 | 0.27 | 0.002 |

**Supplementary Table 5:** Gene set enrichment analysis of all the proteins passing the criteria of p-value <0.05 and Abs FC>1.2) obtained from different comparison.

| **S.No.** | **Category Name** | **Accession ID** | | **No. of regulated proteins** | | | **% of gene hit against total genes** | | | **% of gene hit against total function hits** | |
| --- | --- | --- | --- | --- | --- | --- | --- | --- | --- | --- | --- |
| **Cellular Component** | | | | | | | | | | | |
| 1 | Synapse part | GO:0044456 | | 3 | | | 0.80% | | | 0.40% | |
| 2 | Membrane part | GO:0044425 | | 25 | | | 6.50% | | | 3.30% | |
| 3 | Membrane | GO:0016020 | | 51 | | | 13.20% | | | 6.70% | |
| 4 | Synapse | GO:0045202 | | 3 | | | 0.80% | | | 0.40% | |
| 5 | Organelle part | GO:0044422 | | 64 | | | 16.60% | | | 8.40% | |
| 6 | Extracellular region part | GO:0044421 | | 12 | | | 3.10% | | | 1.60% | |
| 7 | Cell junction | GO:0030054 | | 4 | | | 1.00% | | | 0.50% | |
| 8 | Membrane-enclosed lumen | GO:0031974 | | 29 | | | 7.50% | | | 3.80% | |
| 9 | Protein-containing complex | GO:0032991 | | 72 | | | 18.70% | | | 9.50% | |
| 10 | Supramolecular complex | GO:0099080 | | 5 | | | 1.30% | | | 0.70% | |
| 11 | Extracellular region | GO:0005576 | | 12 | | | 3.10% | | | 1.60% | |
| 12 | Cell | GO:0005623 | | 174 | | | 45.10% | | | 22.90% | |
| 13 | Cell part | GO:0044464 | | 174 | | | 45.10% | | | 22.90% | |
| 14 | Organelle | GO:0043226 | | 132 | | | 34.20% | | | 17.40% | |
| **Biological Processes** | | | | | | | | | | | |
| 1 | Developmental process | GO:0032502 | | 26 | | | 6.70% | | | 3.80% | |
| 2 | Multicellular organismal process | GO:0032501 | | 27 | | | 7.00% | | | 3.90% | |
| 3 | Cellular process | GO:0009987 | | 171 | | | 44.30% | | | 24.70% | |
| 4 | Reproduction | GO:0000003 | | 6 | | | 1.60% | | | 0.90% | |
| 5 | Cell population proliferation | GO:0008283 | | 5 | | | 1.30% | | | 0.70% | |
| 6 | Localization | GO:0051179 | | 42 | | | 10.90% | | | 6.10% | |
| 7 | Reproductive process | GO:0022414 | | 6 | | | 1.60% | | | 0.90% | |
| 8 | Multi-organism process | GO:0051704 | | 6 | | | 1.60% | | | 0.90% | |
| 9 | Biological adhesion | GO:0022610 | | 5 | | | 1.30% | | | 0.70% | |
| 10 | Immune system process | GO:0002376 | | 7 | | | 1.80% | | | 1.00% | |
| 11 | Cellular component organization or biogenesis | GO:0071840 | | 72 | | | 18.70% | | | 10.40% | |
| 12 | Biological regulation | GO:0065007 | | 92 | | | 23.80% | | | 13.30% | |
| 13 | Growth | GO:0040007 | | 1 | | | 0.30% | | | 0.10% | |
| 14 | Signalling | GO:0023052 | | 41 | | | 10.60% | | | 5.90% | |
| 15 | Metabolic process | GO:0008152 | | 112 | | | 29.00% | | | 16.20% | |
| 16 | Response to stimulus | GO:0050896 | | 58 | | | 15.00% | | | 8.40% | |
| 17 | Biological phase | GO:0044848 | | 3 | | | 0.80% | | | 0.40% | |
| 18 | Rhythmic process | GO:0048511 | | 1 | | | 0.30% | | | 0.10% | |
| 19 | Locomotion | GO:0040011 | | 11 | | | 2.80% | | | 1.60% | |
| **Protein class** | | | | | | | | | | |  |
| 1 | Extracellular matrix protein | | PC00102 | | 2 | 0.50% | | | 1.10% | |  |
| 2 | Cytoskeletal protein | | PC00085 | | 11 | 2.80% | | | 6.20% | |  |
| 3 | Transporter | | PC00227 | | 5 | 1.30% | | | 2.80% | |  |
| 4 | Scaffold/adaptor protein | | PC00226 | | 9 | 2.30% | | | 5.10% | |  |
| 5 | Nucleic acid binding protein | | PC00171 | | 24 | 6.20% | | | 13.60% | |  |
| 6 | Intercellular signal molecule | | PC00207 | | 9 | 2.30% | | | 5.10% | |  |
| 7 | Protein-binding activity modulator | | PC00095 | | 17 | 4.40% | | | 9.60% | |  |
| 8 | Calcium-binding protein | | PC00060 | | 1 | 0.30% | | | 0.60% | |  |
| 9 | Gene-specific transcriptional regulator | | PC00264 | | 12 | 3.10% | | | 6.80% | |  |
| 10 | Translational protein | | PC00263 | | 10 | 2.60% | | | 5.60% | |  |
| 11 | Metabolite interconversion enzyme | | PC00262 | | 18 | 4.70% | | | 10.20% | |  |
| 12 | Protein modifying enzyme | | PC00260 | | 34 | 8.80% | | | 19.20% | |  |
| 13 | Chromatin/chromatin-binding, or -regulatory protein | | PC00077 | | 5 | 1.30% | | | 2.80% | |  |
| 14 | Transfer/carrier protein | | PC00219 | | 2 | 0.50% | | | 1.10% | |  |
| 15 | Membrane traffic protein | | PC00150 | | 11 | 2.80% | | | 6.20% | |  |
| 16 | Chaperone | | PC00072 | | 1 | 0.30% | | | 0.60% | |  |
| 17 | Cell junction protein | | PC00070 | | 2 | 0.50% | | | 1.10% | |  |
| 18 | Transmembrane signal receptor | | PC00197 | | 4 | 1.00% | | | 2.30% | |  |
| **Molecular function** | | | | | | | | | | |  |
| 1 | Translation regulator activity | | GO:0045182 | | 2 | 0.50% | | | 0.90% | |  |
| 2 | Transcription regulator activity | | GO:0140110 | | 9 | 2.30% | | | 3.90% | |  |
| 3 | Molecular transducer activity | | GO:0060089 | | 13 | 3.40% | | | 5.70% | |  |
| 4 | Binding | | GO:0005488 | | 95 | 24.60% | | | 41.50% | |  |
| 5 | Structural molecule activity | | GO:0005198 | | 4 | 1.00% | | | 1.70% | |  |
| 6 | Molecular function regulator | | GO:0098772 | | 17 | 4.40% | | | 7.40% | |  |
| 7 | Catalytic activity | | GO:0003824 | | 87 | 22.50% | | | 38.00% | |  |
| 8 | Transporter activity | | GO:0005215 | | 2 | 0.50% | | | 0.90% | |  |
| **Pathways** | | | | | | | | | | | |
| 1 | Axon guidance mediated by netrin | | P00009 | | 2 | 0.50% | | 0.80% | | | |
| 2 | Axon guidance mediated by Slit/Robo | | P00008 | | 3 | 0.80% | | 1.20% | | | |
| 3 | Metabotropic glutamate receptor group III pathway | | P00039 | | 2 | 0.50% | | 0.80% | | | |
| 4 | Axon guidance mediated by semaphorins | | P00007 | | 1 | 0.30% | | 0.40% | | | |
| 5 | Apoptosis signalling pathway | | P00006 | | 4 | 1.00% | | 1.60% | | | |
| 6 | Ionotropic glutamate receptor pathway | | P00037 | | 1 | 0.30% | | 0.40% | | | |
| 7 | De novo purine biosynthesis | | P02738 | | 1 | 0.30% | | 0.40% | | | |
| 8 | Angiogenesis | | P00005 | | 9 | 2.30% | | 3.60% | | | |
| 9 | Interleukin signalling pathway | | P00036 | | 4 | 1.00% | | 1.60% | | | |
| 10 | Alzheimer disease-presenilin pathway | | P00004 | | 2 | 0.50% | | 0.80% | | | |
| 11 | Interferon-gamma signalling pathway | | P00035 | | 2 | 0.50% | | 0.80% | | | |
| 12 | 5HT2 type receptor mediated signalling pathway | | P04374 | | 2 | 0.50% | | 0.80% | | | |
| 13 | Alzheimer disease-amyloid secretase pathway | | P00003 | | 4 | 1.00% | | 1.60% | | | |
| 14 | Integrin signalling pathway | | P00034 | | 12 | 3.10% | | 4.80% | | | |
| 15 | Insulin/IGF pathway-protein kinase B signalling cascade | | P00033 | | 2 | 0.50% | | 0.80% | | | |
| 16 | Insulin/IGF pathway-mitogen activated protein kinase kinase/MAP kinase cascade | | P00032 | | 2 | 0.50% | | 0.80% | | | |
| 17 | Inflammation mediated by chemokine and cytokine signalling pathway | | P00031 | | 10 | 2.60% | | 4.00% | | | |
| 18 | Asparagine and aspartate biosynthesis | | P02730 | | 1 | 0.30% | | 0.40% | | | |
| 19 | Ubiquitin proteasome pathway | | P00060 | | 3 | 0.80% | | 1.20% | | | |
| 20 | Synaptic vesicle trafficking | | P05734 | | 2 | 0.50% | | 0.80% | | | |
| 21 | GABA-B receptor II signalling | | P05731 | | 1 | 0.30% | | 0.40% | | | |
| 22 | Huntington disease | | P00029 | | 4 | 1.00% | | 1.60% | | | |
| 23 | p53 pathway | | P00059 | | 5 | 1.30% | | 2.00% | | | |
| 24 | p53 pathway feedback loops 2 | | P04398 | | 4 | 1.00% | | 1.60% | | | |
| 25 | Heterotrimeric G-protein signalling pathway-Gq alpha and Go alpha mediated pathway | | P00027 | | 6 | 1.60% | | 2.40% | | | |
| 26 | p53 pathway by glucose deprivation | | P04397 | | 1 | 0.30% | | 0.40% | | | |
| 27 | Heterotrimeric G-protein signalling pathway-Gi alpha and Gs alpha mediated pathway | | P00026 | | 8 | 2.10% | | 3.20% | | | |
| 28 | Wnt signalling pathway | | P00057 | | 7 | 1.80% | | 2.80% | | | |
| 29 | Hedgehog signalling pathway | | P00025 | | 2 | 0.50% | | 0.80% | | | |
| 30 | VEGF signalling pathway | | P00056 | | 4 | 1.00% | | 1.60% | | | |
| 31 | Transcription regulation by bZIP transcription factor | | P00055 | | 5 | 1.30% | | 2.00% | | | |
| 32 | Thyrotropin-releasing hormone receptor signalling pathway | | P04394 | | 2 | 0.50% | | 0.80% | | | |
| 33 | General transcription regulation | | P00023 | | 3 | 0.80% | | 1.20% | | | |
| 34 | Toll receptor signalling pathway | | P00054 | | 3 | 0.80% | | 1.20% | | | |
| 35 | Ras Pathway | | P04393 | | 7 | 1.80% | | 2.80% | | | |
| 36 | Adenine and hypoxanthine salvage pathway | | P02723 | | 1 | 0.30% | | 0.40% | | | |
| 37 | T cell activation | | P00053 | | 4 | 1.00% | | 1.60% | | | |
| 38 | P53 pathway feedback loops 1 | | P04392 | | 1 | 0.30% | | 0.40% | | | |
| 39 | FGF signalling pathway | | P00021 | | 12 | 3.10% | | 4.80% | | | |
| 40 | TGF-beta signalling pathway | | P00052 | | 5 | 1.30% | | 2.00% | | | |
| 41 | Oxytocin receptor mediated signalling pathway | | P04391 | | 2 | 0.50% | | 0.80% | | | |
| 42 | FAS signalling pathway | | P00020 | | 2 | 0.50% | | 0.80% | | | |
| 43 | Plasminogen activating cascade | | P00050 | | 1 | 0.30% | | 0.40% | | | |
| 44 | Endothelin signalling pathway | | P00019 | | 3 | 0.80% | | 1.20% | | | |
| 45 | EGF receptor signalling pathway | | P00018 | | 12 | 3.10% | | 4.80% | | | |
| 46 | p38 MAPK pathway | | P05918 | | 2 | 0.50% | | 0.80% | | | |
| 47 | Parkinson disease | | P00049 | | 9 | 2.30% | | 3.60% | | | |
| 48 | DNA replication | | P00017 | | 2 | 0.50% | | 0.80% | | | |
| 49 | PI3 kinase pathway | | P00048 | | 6 | 1.60% | | 2.40% | | | |
| 50 | Cytoskeletal regulation by Rho GTPase | | P00016 | | 1 | 0.30% | | 0.40% | | | |
| 51 | PDGF signalling pathway | | P00047 | | 10 | 2.60% | | 4.00% | | | |
| 52 | Circadian clock system | | P00015 | | 1 | 0.30% | | 0.40% | | | |
| 53 | Oxidative stress response | | P00046 | | 3 | 0.80% | | 1.20% | | | |
| 54 | Cholesterol biosynthesis | | P00014 | | 1 | 0.30% | | 0.40% | | | |
| 55 | Histamine H1 receptor mediated signalling pathway | | P04385 | | 3 | 0.80% | | 1.20% | | | |
| 56 | Notch signalling pathway | | P00045 | | 1 | 0.30% | | 0.40% | | | |
| 57 | Heme biosynthesis | | P02746 | | 1 | 0.30% | | 0.40% | | | |
| 58 | Cell cycle | | P00013 | | 1 | 0.30% | | 0.40% | | | |
| 59 | Succinate to proprionate conversion | | P02777 | | 1 | 0.30% | | 0.40% | | | |
| 60 | Cadherin signalling pathway | | P00012 | | 2 | 0.50% | | 0.80% | | | |
| 61 | Muscarinic acetylcholine receptor 2 and 4 signalling pathway | | P00043 | | 2 | 0.50% | | 0.80% | | | |
| 62 | Dopamine receptor mediated signalling pathway | | P05912 | | 1 | 0.30% | | 0.40% | | | |
| 63 | Muscarinic acetylcholine receptor 1 and 3 signalling pathway | | P00042 | | 1 | 0.30% | | 0.40% | | | |
| 64 | B cell activation | | P00010 | | 7 | 1.80% | | 2.80% | | | |
| 65 | Angiotensin II-stimulated signalling through G proteins and beta-arrestin | | P05911 | | 1 | 0.30% | | 0.40% | | | |
| 66 | Metabotropic glutamate receptor group I pathway | | P00041 | | 1 | 0.30% | | 0.40% | | | |
| 67 | Metabotropic glutamate receptor group II pathway | | P00040 | | 2 | 0.50% | | 0.80% | | | |
| 68 | CCKR signalling map | | P06959 | | 8 | 2.10% | | 3.20% | | | |
| 69 | Gonadotropin-releasing hormone receptor pathway | | P06664 | | 8 | 2.10% | | 3.20% | | | |
| 70 | Toll pathway-drosophila | | P06217 | | 1 | 0.30% | | 0.40% | | | |


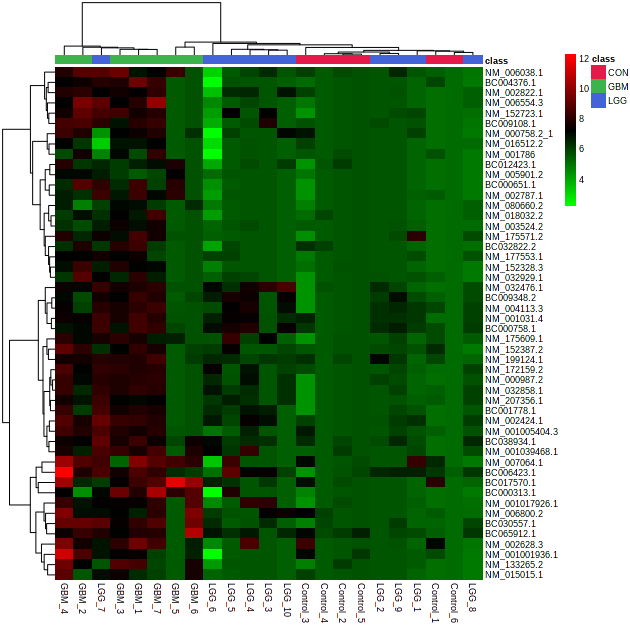


**Supplementary Figure 1:** Unsupervised clustering of the three cohort. The heat-map above depicts that the GBM samples showed an increased number of TA autoantibodies as compared to low grade glioma samples. The GBM cohort (green) segregated together, whereas, a lot of overlap could be seen in LGG and control samples.


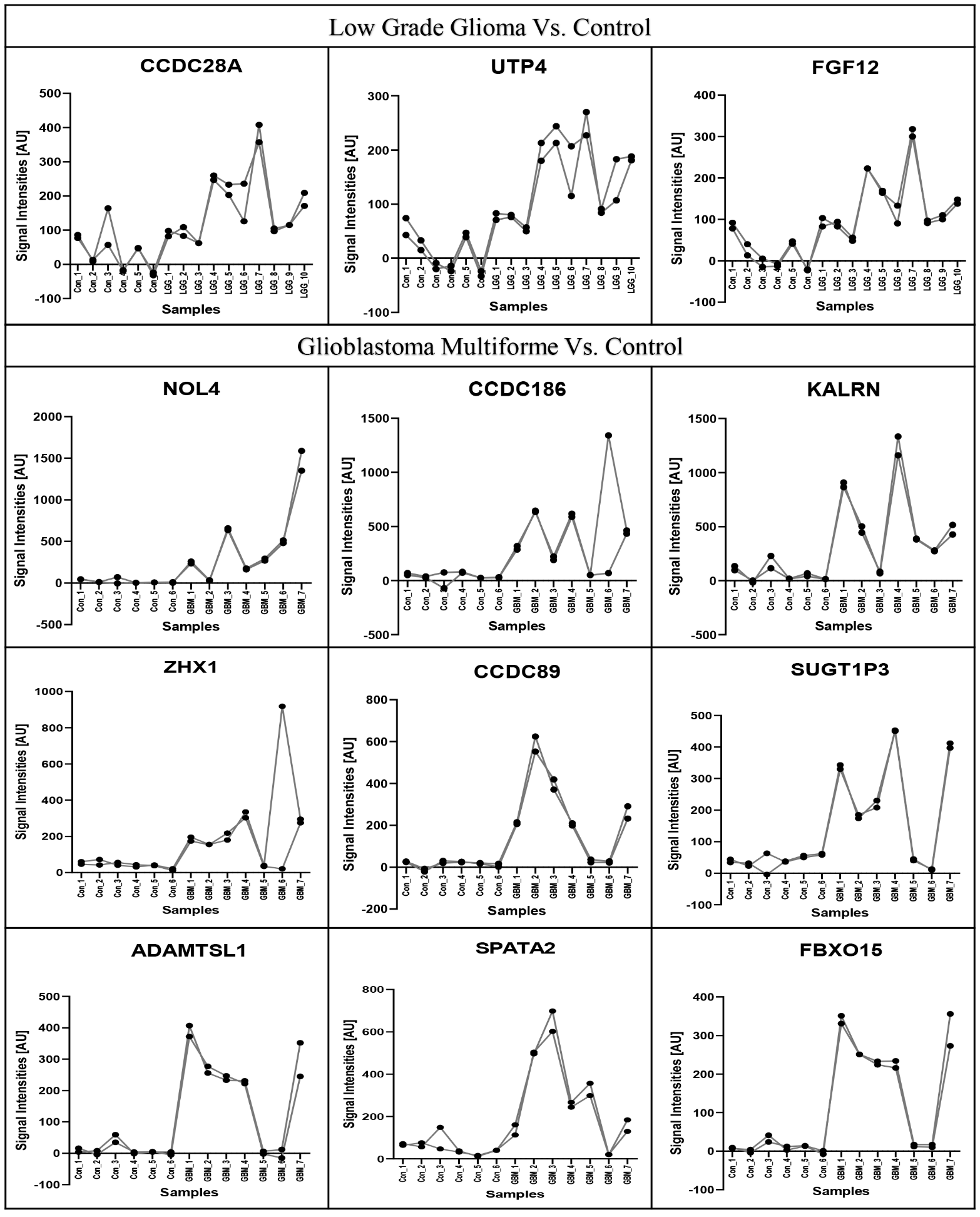


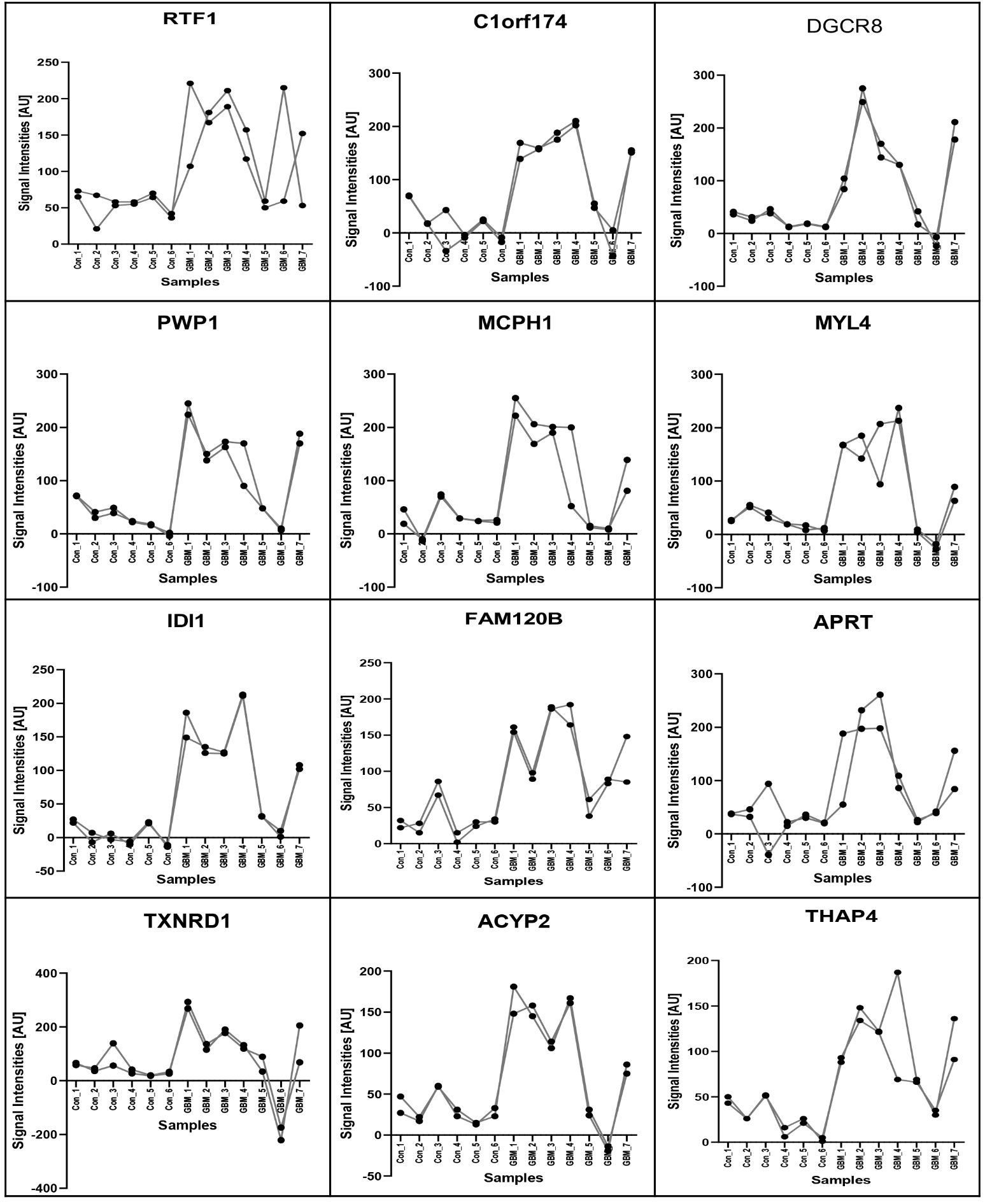


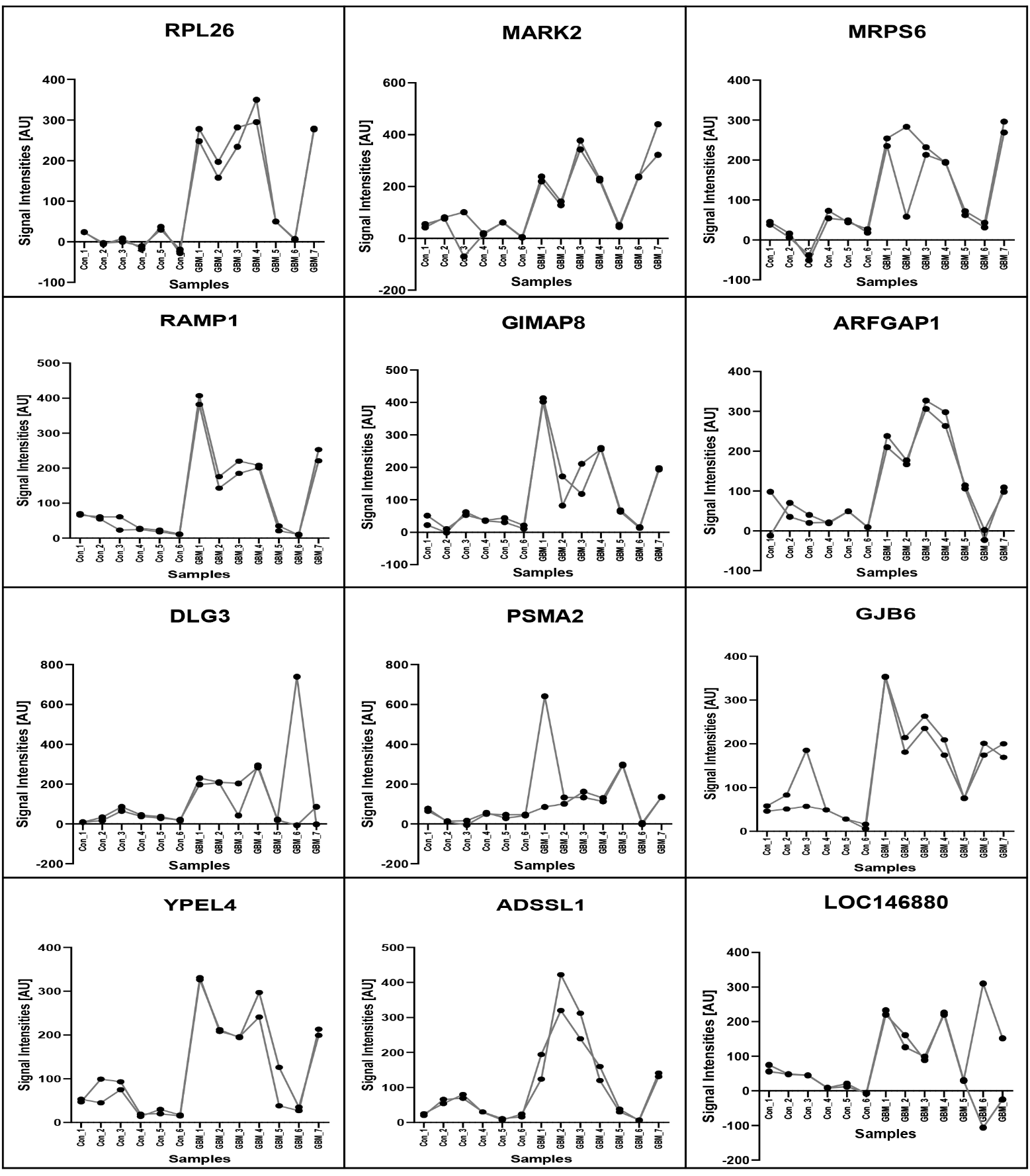


**Supplementary Figure 2:** Dot plots of the potentially antigenic proteins (raw p-value<0.005 and FC>1.2) in LGG and GBM samples. Since each protein spot is printed in duplicate, there are two intensity value (represented by dots) for each sample in the plots.
